# Stress-induced alterations of social behavior are reversible by antagonism of steroid hormones in C57/BL6 mice

**DOI:** 10.1007/s00210-020-01970-7

**Published:** 2020-09-07

**Authors:** Benedikt Andreas Gasser, Johann Kurz, Walter Senn, Genevieve Escher, Markus Georg Mohaupt

**Affiliations:** 1 Department of Clinical Research, University of Bern, 3010 Berne, Switzerland; 2 Intersci Research Association, Karl Morre Gasse 10, 8430 Leibnitz, Austria; 3 Department of Physiology, University of Bern, 3012 Berne, Switzerland; 4 Division of Nephrology/Hypertension, University of Bern, 3010 Berne, Switzerland; 5 Teaching Hospital Internal Medicine, Lindenhofgruppe, 3006 Berne, Switzerland

**Keywords:** Chronic swim test, Stress induction, C57BL/6 mice, CRH (corticotropin-releasing hormone)–ACTH (adrenocorticotropic hormone), Steroid hormones

## Abstract

Various disturbances of social behavior, such as autism, depression, or posttraumatic stress disorder, have been associated with an altered steroid hormone homeostasis and a dysregulation of the hypothalamus–pituitary–adrenal axis. A link between steroid hormone antagonists and the treatment of stress-related conditions has been suggested. We evaluated the effects of stress induction on social behavior in the three chambers and its potential reversibility upon specific steroid hormone antagonism in mice. C57BL/6 mice were stressed twice daily for 8 days by chronic swim testing. Social behavior was evaluated by measuring, first, the preference for sociability and, second, the preference for social novelty in the three-chamber approach before and after the chronic swim test. The reversibility of behavior upon stress induction was analyzed after applying steroid hormone antagonists targeting glucocorticoids with etomidate, mineralocorticoids with potassium canrenoate, and androgens with cyproterone acetate and metformin. In the chronic swim test, increased floating time from 0.8 ± 0.2 min up to 4.8 ± 0.25 min was detected (*p* < 0.01). In the three-chamber approach, increased preference for sociability and decreased preference for social novelty was detected pre- versus post-stress induction. These alterations of social behavior were barely affected by etomidate and potassium canrenoate, whereas the two androgen antagonists metformin and cyproterone acetate restored social behavior even beyond baseline conditions. The alteration of social behavior was better reversed by the androgen as compared with the glucocorticoid and mineralocorticoid antagonists. This suggests that social behavior is primarily controlled by androgen rather than by glucocorticoid or mineralocorticoid action. The stress-induced changes in preference for sociability are incompletely explained by steroid hormone action alone. As the best response was related to metformin, an effect via glucose levels might confound the results and should be subject to future research.

**Electronic supplementary material:**

The online version of this article (10.1007/s00210-020-01970-7) contains supplementary material, which is available to authorized users.

## Introduction

Long disturbances of social behavior, such as autism, depression, or posttraumatic stress disorder (PTSD), have been associated with an altered steroid homeostasis and a dysregulation of the hypothalamus–pituitary–adrenal axis (HPA) (Bondar et al. 2018; Jacobson 2014; Du and Pang 2015; Sriram et al. 2012). Hence, autistic disorders have been associated with HPA dysregulation (Brosnan et al. 2009; Marinović-Curin et al. 2008; Hoshino et al. 1987), given differences of the anatomic structure of the hypothalamus (Bitsika et al. 2014; Hollocks et al. 2014), of the pituitary gland (Brosnan et al. 2009; Hamza et al. 2010; Curin et al. 2003; Iwata et al. 2011; Xu et al. 2015), and of the adrenal gland (Curin et al. 2003; Baron-Cohen et al. 2015; El-Baz et al. 2014; Taylor and Corbett 2014; Ruta et al. 2011; Ingudomnukul et al. 2007; Majewska et al. 2014; Takagishi et al. 2010; Chakrabarti et al. 2009; Knickmeyer et al. 2006). It is generally accepted that stress modulates the CRH (corticotropin-releasing hormone)–ACTH (adrenocorticotropic hormone)–cortisol system, while influencing the disease mechanism (Sapolsky et al. 2000; Dallman 2007; Verbeek et al. 2019; Chrousos 2009; Apple et al. 1993; Gold 2015). Thus, the CRH–ACTH system seems to be involved in different forms of depression (Gold 2015; Checkley 1996). The behavior of social avoidance is a core symptom of all these diseases. Animal models demonstrated a higher level of social avoidance after exposure to stress (Iñiguez et al. 2014). Interestingly, only 10 days of stress were necessary to alter gene induction as associated with glucocorticoid metabolism (Bondar et al. 2018). Chronic stress is generally characterized by a strong stimulation of the central drive combined with a downregulation of its negative feedback upon increased steroid hormone availability; similar processes are also associated with depression (Checkley 1996). Recent discoveries have shown that steroid hormones can indeed exert rapid effects on social behavior (Steinman and Trainor 2010). Steroid hormones allow to regulate behavior in response to sudden and short-lived environmental or social change serving as intermediators (Steinman and Trainor 2010; Ayash et al. 2019).

In contrast, substances affecting androgens, glucocorticoids, and mineralocorticoids have been considered as treatment options for disorders such as autism, depression, or PTSD (Bradstreet et al. 2006; Wink et al. 2017; Aman et al. 2018). However, antagonists of hormones produced by the adrenal gland are rarely considered as potential treatment of disorders with social avoidance, and the respective impact of molecules with inhibitory action on androgens, glucocorticoids, and mineralocorticoids have not been conclusively studied. Thus, we aimed to analyze the effects of stress induction on social behavior and its potential reversibility upon steroid hormone antagonism. Specifically, we first analyzed baseline conditions in mice using the three-chamber approach. Second, we evaluated the effects of stress induction on social behavior. Third, the reversibility of the stress-induced behavioral changes was elucidated using specific steroid hormone antagonism (Popper 1969).

## Material and methods

### Animals

**Fig. 1:**
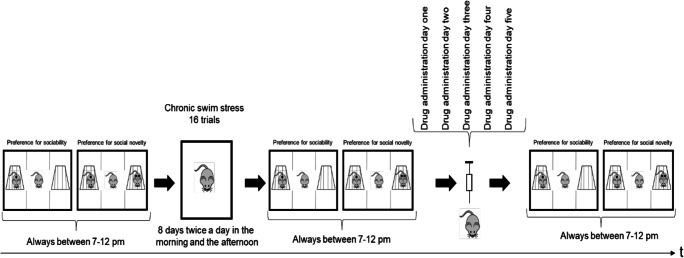
Timeline showing when each of the tests was performed. Mice were assessed before and after the chronic swim test and after drug administration in the three-chamber approach. After the first social testing, chronic Swim stress (8 days of swimming twice a day for 6 min swimming) was employed. Followed by testing in the three-chamber approach the second time. Drug administration was performed on 5 consequent days, followed by last social testing

Ten-week-old female C57BL/6JRccHsd mice all from the same strain were purchased from Envigo Laboratories (Venray, Netherlands). After their arrival, females were placed in IVC cages (501-cm^2^ floor area, Green Line, Tecniplast, Italy) in groups of 6 (except the two female animals under the inverted cup which were housed in separate cages) and maintained under a 12-h dark–light cycle, room temperature in the range of 22 ± 2 °C, and relative humidity in between 45 and 65%. Mice had unrestricted access to irradiated rodent chow diet (3432, Granovit, Switzerland) and autoclaved tap water. Aspen wood bedding (J. Rettenmeier & Söhne GmbH, Germany), paper nestles, and cage enrichment (red PVC house) (Plexx, Netherlands; LAB & VET Service GmbH, Austria) were provided. Mice were not synchronized. Animals in each cage were randomly subjected to the force swim test. The strain of C57BL/6J mice was selected due to their behavioral profile, including moderate to high levels of social approach, exploration, and reversal learning (Moy et al. 2007; Moy et al. 2004). Female mice were used due to easier handling, higher preference for social behavior, and less extraadrenal interference (Bronson 1979; Turcu et al. 2014). Experiments were performed during their active periods after 19:00. Five groups consisting of 8 animals were subjected to chronic swim testing and randomized to the control group or specific drug interventions (Fig. 1). The animal experiments were approved by the Ethics Committee for Animal Experiments of the Veterinary Administration of the Canton of Bern, Switzerland (BE128/16), and conformed to the rules of the Swiss Federal Act on Animal Protections. Experiments were carried out at the central animal facility of the University of Bern.

### Chronic swim stress

We chose forced swimming as behavioral mouse model of depression or autism, which is stress response-based (Brown et al. 2001; Steru et al. 1985; Weiss et al. 1975; Blanchard et al. 1993; Seligman and Maier 1967). Distinct behavioral patterns can be discriminated and quantified to identify the maximal disappointment and signs of behavioral depression (Brown et al. 2001). Mice were dropped into a cylinder (height 25 cm, diameter 10 cm, 6 cm of water at 21–23 °C) for 6 min with the duration to immobility being scored (Brown et al. 2001) as adapted in Stone and Lin (2011) (Stone and Lin 2011; Porsolt et al. 1977; Sun and Alkon 2003). Another approach consists of swimming mice daily in lukewarm water in a plexiglass cube 24 × 43 × 23 cm *w* × *h* × *l* for 15 min/day for 4 days, and thereafter once a week. This procedure produced a progressive decrease in distance swum and a concomitant increase in immobility (floating) in about 70% of mice, which persisted unaltered for weeks (Stone and Lin 2011). The swim test seems to be a valid opportunity and an accepted model, which can be easily handled and is generally used for inducing stress in mice (Brown et al. 2001; Yankelevitch-Yahav et al. 2015).

In our setting, the swimming area was 70 × 40 × 15 cm. The water temperature was kept constant at around 24 °C. Immobility was assigned when a mouse was immobile without a forward movement, had ceased to struggle and remained floating motionless with only the finest movements to keep equilibrium and its head out of the water. All experiments in the forced swim test were observed and measured by the same person.

### Three-chamber approach

**Fig. 2:**
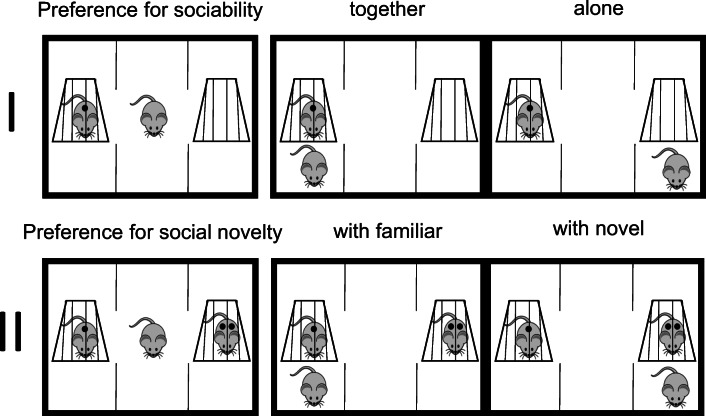
Scheme of the three-chamber approach: a test mouse was first placed in the middle chamber and allowed to explore all three chambers during 5 min for habituation. Afterwards, the first (I) preference for sociability was analyzed by measuring time “together” (time in the same compartment) and time “alone” (in the compartment without a mouse). Time spent is not an assessment of contact but simply the time spent in a respective compartment. Secondly (II), for another 5 min, the preference for social novelty was analyzed by measuring the time spent with the mouse from the previous experiment (“with familiar”) or with a new unfamiliar mouse (“with novel”)

Mice were assessed before and after the chronic swim test and after drug administration in the three-chamber approach. The preference for sociability and for social novelty was analyzed (Moy et al. 2007; Moy et al. 2004) (Fig. 2). In both experimental settings, the mice were initially placed into the center of a 41 × 60 × 28 cm three-chamber apparatus, whereby the outer compartment contained two empty, inverted wire cups. For habituation, the sliding doors were opened and each mouse was allowed to explore all three chambers for 5 min. The mice were returned to the middle of the apparatus, the sliding doors were closed, and companions were placed into the other chamber(s). The doors were released for 5 min to analyze the preference for sociability and the preference for social novelty. The experiment for preference of sociability measured the time shared with a previously unknown companion (“*together*”) versus the time spent alone (“*alone*”). The preference for social novelty experiment measured the time spent with a novel (“*with novel*”) versus the time spent with the previous companion from the sociability experiment (“*with familiar*”) (Fig. 2). The companions in the first sociability experiment and the novel companions in the social novelty experiment were C57BL/6 mice that were always housed separately and had no previous contact with the tested mice. The test material was cleaned with water and dried with paper towels in between experiments; at the end of a test battery, the apparatus was cleaned with ethanol. All experiments in the three-chamber approach were performed by the same person.

### Drug administration

To test the reversibility of stress-induced behavioral changes, drugs were administered to the interventional groups, yet not to the animals under the inverted cup (Fig. 2). Drugs were from Sigma Aldrich (Switzerland), dosing was adapted to average mice weight (19.8 ± 1.02 g) (Brown et al. 2001). (i) Etomidat Lipuro is a galenic solution which was injected intravenous into the tail vein (250 μL, 25 mg/kg) (Anon 2017). Etomidate is an imidazole-based sedative hypnotic whereby lower concentrations are associated with an outlasting effect on adrenal cortical suppression with a mainly anti-glucocorticoid action (Pejo et al. 2012). Etomidate binds with a high affinity to the cytochrome P450 enzyme 11β-hydroxylase and inhibits the enzyme’s function, which converts 11-deoxycortisol to corticosterone inhibiting glucocorticoid action in general while potently suppressing adrenocortical steroids for a substantial time (more than 5 h) (de Jong et al. 1984; Wagner et al. 1984). (ii) The competitive mineralocorticoid receptor antagonist potassium canrenoat (Canrenon) was sterilized by microfiltration and concentrated (1.25 mg/kg in 125 μL subcutaneously) (Anon 2017; Cunningham et al. 2012). (iii) The androgen receptor antagonist cyproteronacetat was sterilized and diluted with castor oil plant (sterilized at 180 °C for 30 min, 6.25 mg/kg in 125 μL subcutaneously) (Anon 2017; Kolkhof and Bärfacker 2017). (iv) Metformin inhibits testosterone synthesis and was pestled and soluted in Ringer lactate (5.00 mg/kg in 125 μL subcutaneously) (Anon 2017; Ozaki et al. 2006).

### Statistical analysis

For each trial in the chronic swim test, the mean ± SEM of seconds floating was calculated. Linear regression between the mean and the number of trials was calculated. From testing in the three-chamber approach, the mean ± SEM of the time spent in a respective chamber was computed. All measurements underwent the Kolmogorov–Smirnov testing for normal distribution. As normality distribution could not be rejected for subsamples of an alpha-level of 0.1, two-sided heteroscedastic *t* tests were performed, which were corrected for multiple comparison with Bonferroni. The calculations were made with GraphPad Prism 5.0 (GraphPad Software, Inc., La Jolla, CA, USA).

## Results

**Fig. 3:**
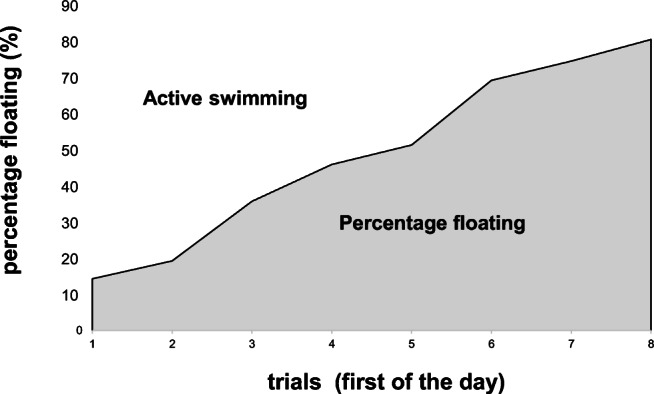
Effects of chronic swim stress on floating time. The number of swimming trials (first of the day) is on the *x*-axis and the time that was floated with the head out of the water without movement in percentage is on the *y*-axis

The results of chronic swim testing are given in Fig. 3. A steady increase in floating was detected over time with flow time from 0.8 ± 0.2 min up to 4.8 ± 0.3 min (*p* < 0.01) of the 6 min total time (all: linear regression 0.3030 * *n* + 0.523, *R*^2^ = 0.9614 and first daily trial: linear regression 0.6107 * *n* + 0.2139, *R*^2^ = 0.9825).

**Fig. 4:**
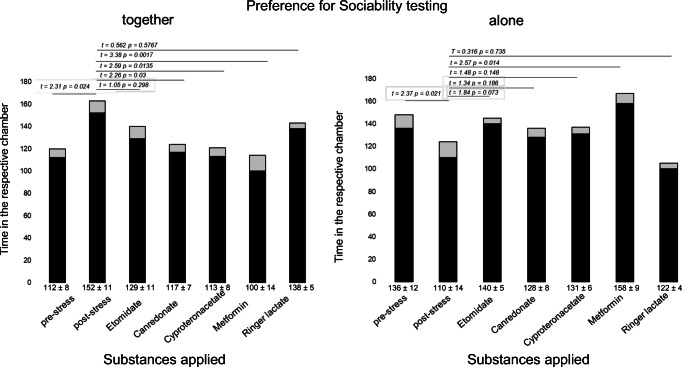
Time measured in the three-chamber approach. Pre-stress, post-stress, and after drug administration. The extension of “together” was reversed upon all pharmacological interventions. *N* = 40 for pre-stress, and *N* = 8 for post-stress. Data are given as the mean ± SEM. Df pre versus post is 78; df post-stress versus application with one of the drugs is 46. In order to get a proxy post application, *p* values according to Bonferroni correction should be multiplied by four

**Fig. 5:**
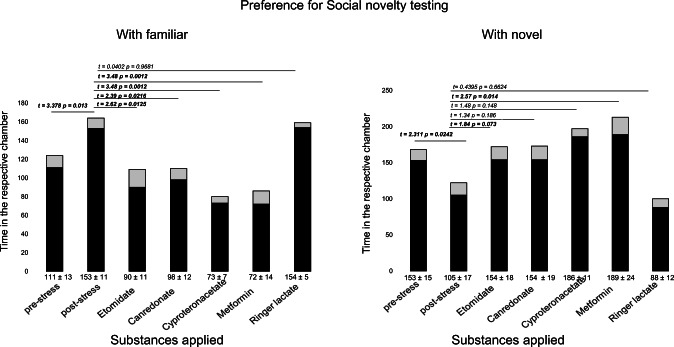
Time measured in the three-chamber approach. Pre-stress, post-stress, and after drug administration. The antiandrogen effect of cyproterone acetate and metformin increased preference for social novelty beyond baseline conditions. *N* = 40 for pre-stress, and *N* = 8 for post-stress. Data are given as the mean ± SEM. Df pre versus post is 78; df post-stress versus application with one of the drugs is 46. In order to get a proxy post application, *p* values according to Bonferroni correction should be multiplied by four

Figures 4 and 5 summarize the effects pre- versus post-swimming and after drug administration. The first column shows the baseline condition, and the second column shows the measurements after stress induction but prior to drug administration. The alterations of time spent in each of the four conditions after stress induction were always significant. Stress significantly increased “together” and decreased “alone,” implying that stress increases the preference for sociability (Fig. 4).

In the preference for social novelty, there was an increase with “familiar” and a decrease “with novel,” implying a reduced preference for social novelty after stress induction (Fig. 5).

The drug administration of the four substances yielded partly to the reinforcement of pre-stress conditions. However, etomidate with mainly glucocorticoid action is only restored in the preference for the social novelty experiment. Potassium canrenoate with mainly mineralocorticoid action yielded no significant change of preference for sociability, but a significant decrease “with familiar” and an increase “with novel” resulting in the preference for social novelty experiment. Cyproterone acetate significantly decreased in “together” but had no significant effect on “alone.” In the preference for social novelty, a significant effect on restoring above baseline conditions was measured. Finally, metformin was the only tested substance that affected the preferences for sociability and social novelty significantly.

## Discussion

In these experiments, we could clearly establish the impact of induced stress using the forced swimming model on social behavior with an increase of the preference for sociability and a decrease in the preference of social novelty. In line with our hypothesis of an overly androgenic drive during these exposures being responsible for these behavorial changes, drugs with antiandrogenic action increased the preferences for sociability and social novelty back to baseline. In contrast, the glucocorticoid antagonist etomidate and the mineralocorticoid antagonist potassium canrenoate had a profoundly reduced effect. The most consistent beneficial response was seen with metformin. It is known that metformin inhibits the mitochondrial 17,20-hydroxylase activity responsible to support androgen production and accordingly significant effects on behavior.

These findings are limited by the fact that metformin is also a potent antidiabetic drug, which might interact with blood sugar levels confounding behavioral responses. Furthermore, measurements of steroid hormones in mice pre versus post-stress were not performed. In consequence, the overall link between induced stress, altered HPA axis, and steroid hormone metabolism remains vague. Other limitations include the fact that the forced swim test was originally developed to modulate learned helplessness (Steru et al. 1985; Seligman and Maier 1967; Stone and Lin 2011; Maier and Seligman 1976). Consequently, the detected results of an increase of floating might also be due to an adaptive strategy instead of a stress response normally occurring in all chronic stress models (Berton et al. 2006; Tsankova et al. 2006; Willner 1997). Yet, increased steroid hormones after stress induction have been described and provide the physiological basis our hypothesis was created upon (Markou et al. 2015). The strength of the induced stress might be tremendous and not just mild. Furthermore, the forced swimming test was also developed as “a new behavioral method to induce a depressed state in mice” which then would link to a depressive behavior (Brown et al. 2001; Stone and Lin 2011). The exact discrimination as in human illness which is characterized by not only social impairment but also other clinical signs, such as insomnia, changes in eating patterns, or cognitive symptoms, is difficult in our experimental setup and could not be captured by the study (Kammerer et al. 2018). As these experiments monitor drug responses, simply dissimilar pharmacodynamics may support unexplainable variance and simply not all mice even from the same strain do react in a similar manner (Krishnan et al. 2007; Touma et al. 2008). Female mice were used for several reasons, yet the ovary (estrous cycle) was not controlled (Bronson 1979; Turcu et al. 2014). Yet, given the clear results, a sustained impact of this variable appears unlikely.

Our result that chronic swim stress elicited not only an increase in social preference but also a decrease in social preference for a novel peer is in contrast to other findings implying the detection of a decrease in preference for sociability and novelty (Toth and Neumann 2013; Blanchard et al. 2003), which could be due to the amount of stress experienced by the animals (Willner et al. 1987).

In human clinical disease, in several diseases such as depression, autism, or PTSD, a dysregulation of HPA seems to exist. Our own data suggest that autistic children exhibited an increased DHEA/cortisol ratio implying a redirection of steroid hormone metabolism towards DHEA via increased 17,20-hydroxylase activity. Feedback-controlled lower cortisol levels will then require further stimulation of the HPA axis even further increasing androgen generation (Gasser et al. 2020; Gasser et al. 2019).

In conclusion, stress-induced alterations of steroid hormone balance do have a modulating impact on social behavior. Upon pharmacological intervention which modulates the steroid hormone availability, this effect was adapted. The major improvement of androgen inhibition was an increased preference for social novelty. Given the induced changes by our intervention and the restoration by reducing androgen responsiveness, we suggest this as a model for autism and an appropriate therapeutic intervention. Of interest, others suggested such an approach even without our data basis. Bradstreet et al. proposed spironolactone for autistic disorders, and metformin was considered in the treatment of autistic disorders and for depression an involvement of the CRH–ACTH system was implied (Gold 2015; Checkley 1996; Bradstreet et al. 2006; Wink et al. 2017; Aman et al. 2018). Given the safety profile for both metformin and spironolactone, a clinical approach might be feasible in human studies. Nevertheless, many factors remain to be elucidated.

## Electronic supplementary material

ESM 1 (XLSX 48 kb)

## Data Availability

All data was generated in house, and no paper mill was used whereby data can be made accessible if requested.
